# Deregulated Adhesion Program in Palatal Keratinocytes of Orofacial Cleft Patients

**DOI:** 10.3390/genes10110836

**Published:** 2019-10-23

**Authors:** Aysel Mammadova, Carine E.L. Carels, Jie Zhou, Christian Gilissen, Maria P.A.C. Helmich, Zhuan Bian, Huiqing Zhou, Johannes W. Von den Hoff

**Affiliations:** 1 Department of Dentistry, Section Orthodontics and Craniofacial Biology, Radboud Institute for Molecular Life Sciences (RIMLS), Radboud University Medical Center, P.O. Box 9101, 6500 HB Nijmegen, The Netherlands; aysel_m@live.com (A.M.);; 2 Department of Human Genetics, KU Leuven, 3000 Leuven, Belgium; carine.carels@uzleuven.be; 3 Department of Oral Health Sciences, KU Leuven, 3000 Leuven, Belgium; 4 The State Key Laboratory Breeding Base of Basic Science of Stomatology (Hubei-MOST) and Key Laboratory of Oral Biomedicine Ministry of Education, Wuhan University, Wuhan 430079, China; zjhappy1226@hotmail.com (J.Z.);; 5 Department of Human Genetics, Radboud University Medical Center, P.O. Box 9101, 6500 HB Nijmegen, The Netherlands; christian.gilissen@radboudumc.nl (C.G.); jo.zhou@radboudumc.nl (H.Z.); 6 Department of Molecular Developmental Biology, Radboud Institute for Molecular Life Sciences (RIMLS), P.O. Box 9101, 6500 HB Nijmegen, The Netherlands

**Keywords:** craniofacial anomalies, gene expression, molecular biology, cell biology, craniofacial biology/genetics

## Abstract

Orofacial clefts (OFCs) are the most frequent craniofacial birth defects. An orofacial cleft (OFC) occurs as a result of deviations in palatogenesis. Cell proliferation, differentiation, adhesion, migration and apoptosis are crucial in palatogenesis. We hypothesized that deregulation of these processes in oral keratinocytes contributes to OFC. We performed microarray expression analysis on palatal keratinocytes from OFC and non-OFC individuals. Principal component analysis showed a clear difference in gene expression with 24% and 17% for the first and second component, respectively. In OFC cells, 228 genes were differentially expressed (*p* < 0.001). Gene ontology analysis showed enrichment of genes involved in β1 integrin-mediated adhesion and migration, as well as in P-cadherin expression. A scratch assay demonstrated reduced migration of OFC keratinocytes (343.6 ± 29.62 μm) vs. non-OFC keratinocytes (503.4 ± 41.81 μm, *p* < 0.05). Our results indicate that adhesion and migration are deregulated in OFC keratinocytes, which might contribute to OFC pathogenesis.

## 1. Introduction

Orofacial clefts (OFCs) are among the most frequent congenital anomalies with a prevalence of about 8 per 10,000 live births worldwide [1]. OFCs not only cause functional problems pertaining to feeding, hearing, speech and dental functioning, but also negatively affect facial appearance, psychosocial wellbeing and social integration. The majority of OFCs occur as an isolated disorder, however OFCs can also be part of a syndrome [2]. Genetic as well as environmental factors contribute to the aetiology of OFC [2].

OFC is caused by a developmental failure in the fusion of the primary palate (cleft lip and cleft primary palate), the *n* secondary palate (cleft secondary palate) or other areas of the face. Palatogenesis takes place between the 6th and 12th week of human development and involves subsequent outgrowth, elevation, adhesion and fusion of the palatal shelves followed by removal of the medial edge epithelial seam (MES) [3]. Studies in mouse models show that clefts develop following (1) failure of palatal shelf outgrowth, (2) fusion of a palatal shelf with the tongue or mandible, (3) failure of palatal shelf elevation, (4) failure of the palatal shelves to adhere to each other and (5) persistence of the MES [4]. The coordinated regulation of cellular functions, such as proliferation, differentiation, adhesion, migration and apoptosis, is vital for palatogenesis [3]. Proliferation is essential for palatal shelf outgrowth. Differentiation, migration, adhesion and apoptosis are required for fusion of the palatal shelves. A failure of the palatal shelves to adhere, as well as premature adhesion of the palatal shelves to the mandible, the tongue or the oral epithelium can impede proper fusion of palatal shelves end lead to a cleft palate. The embryonic oral cavity is lined with the periderm, a single layer of flattened epithelial cells covering the embryonic epithelia [5], which provides a non-sticking barrier to prevent aberrant epithelial adhesions [3]. Prior to adhesion of the palatal shelves, periderm cells disappear through desquamation [6,7] and possibly also through migration to the oral and nasal side of the palatal shelves [8,9] exposing the medial edge epithelium (MEE). Following epithelial cell intercalation, the MES cells disappear by extrusion and possibly apoptosis allowing the formation of a continuous palate [8,9,10]. Current evidence confirms that periderm cell migration prior to MEE cell adhesion is crucial for successful palatogenesis [8,9]. Hence, disruption in either or both of these processes can lead to a cleft palate. 

Optimal cell migration is characterized by the intermediate state of cellular adhesion. At a low adhesive state, cytoskeletal forces easily break the cell–ECM attachment so that cells fail to generate the traction required for locomotion, whereas at a high adhesive state, cytoskeletal forces are not strong enough to break the cell–ECM attachment [11]. At an intermediate adhesive state, cytoskeletal forces are in balance with adhesion so that traction can be maintained at the front, while it can be disrupted at the rear of the cell, allowing forward cell body movement [11]. In other words, cells are unable to migrate if they are either too strongly attached or not attached to the ECM, requiring intermediate adhesion for optimal migration.

In vitro studies demonstrate that human palatal fibroblasts from OFC patients exhibit an abnormal phenotype with respect to ECM synthesis, expression of TGFβ isoforms and response to retinoic acid compared with non-OFC palatal fibroblasts [12,13,14]. Fibroblasts from a subpopulation of OFC patients also have a faster migration rate in a scratch assay [15]. However, studies on differences between non-OFC vs. OFC palatal keratinocytes are lacking.

We hypothesized that genes related to crucial cellular processes for palatogenesis, such as proliferation, migration, adhesion, differentiation and apoptosis, are deregulated in keratinocytes derived from OFC patients. To test our hypothesis, we firstly performed microarray analysis on palatal keratinocytes obtained from OFC patients (further referred to as OFC keratinocytes) and non-OFC individuals (further referred to as non-OFC keratinocytes). Gene ontology (GO) analysis revealed enrichment of genes involved in adhesion and migration. The expression of these genes was then validated with qPCR in both affected and unaffected cells. Finally, our scratch assay confirmed that migration of OFC keratinocytes was reduced in comparison with non-OFC keratinocytes.

## 2. Materials and Methods

### 2.1. Subjects and Tissue Sampling

The OFC group consisted of 15 children, 10 males and 5 females (aged 1.5 ± 0.2 years), with non-syndromic cleft palates (with or without a cleft lip and alveolus). The OFC group contained 5 subjects with cleft palates only and 10 subjects with a cleft lip and palate. The non-OFC control group consisted of 7 males and 8 females (aged 1.9 ± 0.4 years) without congenital malformations. All subjects gave their informed consent. In the OFC group, 3 mm biopsies of the hard palate mucosa were taken about 1 cm from the medial edge of the cleft during the primary surgical closure. In the non-OFC group, a similar biopsy was taken during a tonsillectomy at about 1 cm from the midline of the hard palate. Palatal keratinocytes were cultured from the biopsy specimens according to a previously reported method [16]. The study protocol was approved by the Central Ethical Committee of The Netherlands (Poz.o544C).

### 2.2. Cell Culture

For the microarray assay, palatal keratinocytes (passage 3) were thawed and cultured in T75 flasks in 10 mL keratinocyte serum-free medium (K-SFM) (Gibco, Invitrogen, 10744019) with supplements and incubated at 37 °C. Once the cells reached 80–90% confluence, they were cultured for an additional 24 h in fresh medium. Subsequently, the cells were washed with cold phosphate-buffered saline (PBS), lysed in RLT buffer (RNeasy Mini Kit^®^) containing β-mercaptoethanol, and stored at −80 °C. 

### 2.3. RNA Isolation and Microarray Expression Analysis

Total RNA was extracted using the RNeasy Mini Kit^®^ (Qiagen, 74104, Hilden, Germany) according to the manufacturer’s protocol and quantified spectrophotometrically. Sample quality was analysed using the Agilent 2100 Bioanalyzer^®^ (Agilent Technologies, Santa Clara, CA, USA). RNA was pooled randomly into 5 pools of 3 non-OFC or 3 OFC subjects. Each pool contained the same amount of RNA from each cell line. A total of 6 μg RNA from each pool was used for cDNA synthesis. A total of 10 μg labelled cDNA was hybridized to the Human Genome U133 Plus 2.0 Array^®^ (Affymetrix, Santa Clara, CA, USA) according to the manufacturer’s protocol at the Department of Human Genetics, Radboud Institute of Molecular Sciences, Nijmegen, The Netherlands. Arrays were scanned with a GeneChip^®^ Scanner 3000 (Affymetrix). 

Affymetrix CEL files were imported into Affymetrix Expression Console version 1.1. Control probes were subsequently extracted using the default RMA algorithm for quality analysis. The area under the curve (AUC) of the receiver operator characteristic was calculated using the positive and negative control probes. All arrays had an AUC score above the empirically defined threshold of 0.85 indicating a good separation of the positive controls from the negative controls. Subsequently, CEL files were imported into Partek (Partek Genomic Suite software, version 6.4; Partek Inc.) where only core exons were extracted and normalized using the RMA algorithm with GC background correction. Core transcript summaries were calculated using the mean intensities of the corresponding probe sets [17]. Principal component analysis was used as quality control and was performed with the python package scikit-learn, version 0.17.1 (http://scikit-learn.org). Samples were divided in two groups, OFC patient cells and non-OFC controls. A paired t-test was performed on the ^2^log intensities. The fold changes between OFC and non-OFC samples were calculated from these data. Gene Ontology (GO) analysis was performed on differentially regulated genes (*p* < 0.001) using the DAVID Bioinformatics Resources website [18,19], and genes were further prioritized using known adhesion genes (*CDH3*, *FBLIM1*, *ITGB1*, *LPP*, *MLLT4*, *ARHGAP26*, *SMAD7* and *TNS3*) as the training set in Endeavour software. Therefore, the final gene list also contains genes with a *p*-value > 0.001. Expression differences for genes of interest were subsequently validated using qPCR.

### 2.4. Reverse Transcription and Real-Time Quantitative PCR

cDNA was synthesized from 1 μg total RNA of the individual cell lines using the SuperScript^®^ II Reverse Transcriptase (Invitrogen, 18064022, Carlsbad, CA, USA) according to the manufacturer’s protocol and followed by digestion with the Deoxyribonuclease I, Amplification Grade^®^ (DNase I, Amp Grade) (Invitrogen, 18068015). Real-time quantitative PCR (qPCR) was performed in duplicate in a total reaction volume of 25 μL containing 12.5 μL SYBR Green Supermix^®^ (Bio-Rad Laboratories, 1708885, Hercules, CA, USA), 5 μL of cDNA, 4.5 μL of RNAse-free water, 3 μL of 2.5 μM forward primer and 3 μL of 2.5 μM reverse primer. All primers were designed with exon spanning wherever possible and were obtained from Biolegio^®^ (Nijmegen, the Netherlands). Primer sequences are provided in Supplementary Table S1. The cDNA amount was amplified in the C1000 Thermal Cycler^®^ (Bio-Rad Laboratories), and fluorescence was analysed using the CFX96 IVD Real-Time PCR System^®^ (Bio-Rad Laboratories). The PCR conditions were one cycle at 95 °C for 3 min, followed by 39 cycles of 95 °C for 15 s and 60 °C for 30 s, and finally a temperature increase from 65 °C to 95 °C at 0.5 °C intervals. The gene expression was normalized to that of *human acidic ribosomal protein P0 (hARP)* and presented as 2^−ΔCt^. We used *hARP* as a housekeeping gene because it is more stable in keratinocytes than *actin* or *GAPDH* [20].

Initial statistical analysis revealed that the expression values of two non-OFC (C4 and C13) and two OFC (H196 and H247) cell lines deviated more than two standard deviations from the mean for the majority of the genes. Therefore, these four cell lines were excluded for the final analysis. The expression values of *CDH3* and *THBS1* were ^10^log transformed because of non-normal distribution. The difference between the expression of the candidate genes in OFC cells and non-OFC cells was tested with an unpaired t-test. A value of *p* < 0.05 was considered significant.

### 2.5. Immunostaining of Tissue Samples

Tissue samples were fixed for 4 h in 4% paraformaldehyde in 0.1 M phosphate buffer at room temperature and embedded in paraffin. Serial sections (5 µm) were cut, mounted on Superfrost-Plus slides (Menzel-Gläser), deparaffinated and rehydrated. Immunohistochemical staining was performed using the avidin–biotin–peroxidase complex method. For the β1 integrin staining, sections were pre-treated with 0.1% trypsin (Sigma-Aldrich, T4799-25G) in PBS for antigen retrieval. For the P-cadherin staining an additional microwave treatment in citrate buffer was performed. Sections were then incubated with a mouse monoclonal 1-integrin antibody (1:100)) (Santa Cruz, Dallas, Texas, USA, sc9970) or a rabbit polyclonal P-cadherin antibody (1:400) (Abcam, Cambridge, UK, ab190076). Then the sections were incubated with a biotinylated secondary antibody (1:500) (Jackson ImmunoResearch, Cambridgeshire, UK). Subsequently, the avidine–biotin complex was applied. Finally, the sections were stained with a di-amino benzidine solution (Sigma-Aldrich, D5637-1G) and counterstained with Mayer’s haematoxylin.

### 2.6. Scratch Assay

Palatal keratinocytes (*n* = 15 for both OFC and non-OFC samples) were cultured in triplicate in 24-well flat bottom plates (1 × 10^5^ cells/well) and incubated at 37 °C. When cells reached confluence, the monolayer was scratched with a p-200 pipette tip to create two uniform cell-free lines of 843 ± 9 μm width in each well crossing each other at a 90° angle. The 0 h scratch width was determined by averaging 10 separate cultures of non-OFC cells after staining. After removal of the detached cells by gently washing with PBS in the experimental cultures, the adherent cells were incubated in K-SFM without supplements for 16 h to observe migration. After 16 h, the cells were washed with PBS three times, and fixed with 4% paraformaldehyde at room temperature for 30 min. Then cells were washed three times with ddH_2_O and stained with 0.1% crystal violet at room temperature for 30 min. The cells were washed again three times with ddH_2_O and were dried in the hood. The width of the scratches was measured at 12 predetermined locations (6 locations on each scratch line) in each well using a transparent indicator sheet. Photographs were made with a Leica MZ12 microscope^®^ (Leica Microsystems, Wetzlar, Germany) equipped with a digital camera. 

The mean migration distance was determined for each well and the mean with standard deviation was calculated for the three replicate wells. The difference between the original width of the scratch and the width after 16 h was expressed in μm. The mean migration was calculated as the mean difference between the original width of the scratch and the width after 16 h. Subsequently, the difference between OFC and non-OFC samples was tested by an unpaired t-test. A value of *p* < 0.05 was considered significant. 

## 3. Results

### 3.1. Microarray Analysis 

**Figure 1:**
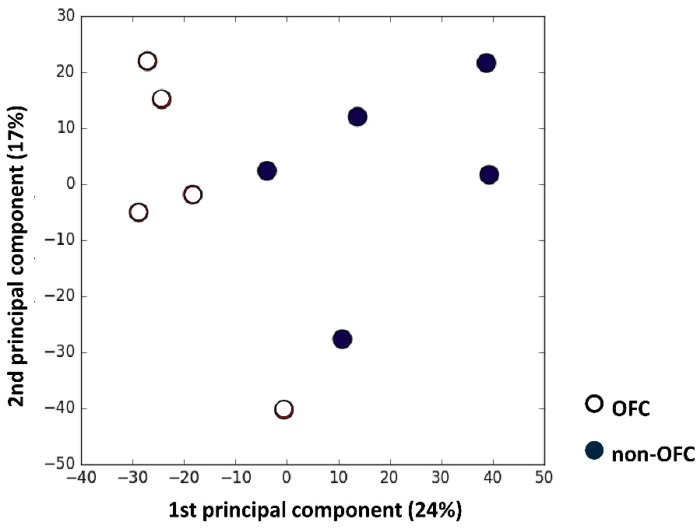
PCA analysis of microarray data from orofacial cleft (OFC) and non-OFC cells. The first and second component were responsible for 24% and 17% of the difference respectively.

The Human Genome U133 Plus 2.0 Array (Affymetrix) covering over 47,000 transcripts has been used for the microarray analysis. PCA analysis showed a clear difference in gene expression between the two cell types, with 24% and 17% for the first and second component, respectively (Figure 1). Using the significance of differentially regulated genes (*p* < 0.001), 228 genes were identified, and among them, 50 genes were upregulated and 178 genes were downregulated in OFC keratinocytes vs. non-OFC keratinocytes.

### 3.2. Gene Ontology Analysis

In order to analyse the functional annotation of the 228 differentially expressed genes, we performed Gene Ontology (GO) analysis using the DAVID Bioinformatics Resources website [18,19]. GO analysis showed an enrichment of genes related to adhesion and migration (Supplementary Table S1). Next, to identify more genes involved in adhesion and migration in our dataset, we used Endeavour software [21,22] and a panel of known adhesion genes (*CDH3*, *FBLIM1*, *ITGB1*, *LPP*, *MLLT4*, *ARHGAP26*, *SMAD7* and *TNS3*) as the training set to prioritize and expand the gene list. This analysis resulted in 21 genes related to adhesion (*ARHGAP26, CDH3, DOCK5, DST, EGFR, FBLIM1, ITGA3, ITGB1, ITGB4, JAK1, LAMA3, LAMC1, LPP, MICAL2, MLLT4, PARVA, PXN, SMAD7, THBS1, TNC* and *TNS3*), all downregulated in OFC keratinocytes vs. non-OFC keratinocytes. Table 1 summarizes the statistical analysis and the raw array data of these genes. 

### 3.3. Validation of the Prioritized Genes

**Figure 2:**
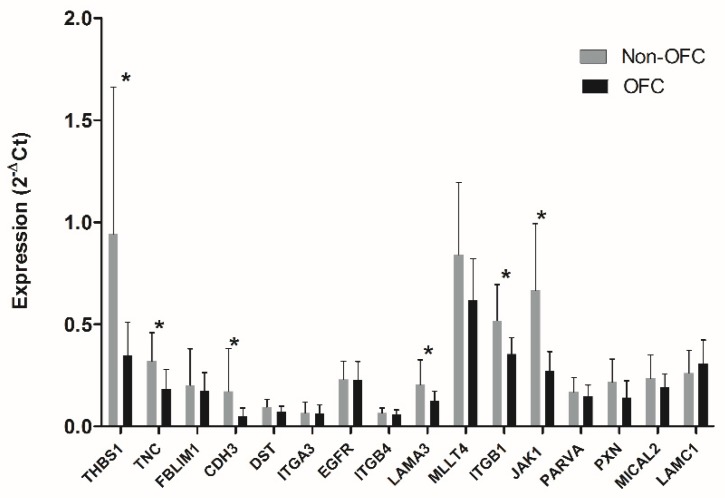
Expression of adhesion genes in the individual cell lines analysed by qPCR. The expression of *CDH3, ITGB1*, *JAK1*, *LAMA3, THBS1* and *TNC* is significantly downregulated in OFC keratinocytes vs. non-OFC keratinocytes (*, *p* < 0.05).

Among the final 21 genes, 4 genes (*ARHGAP26, DOCK5, SMAD7* and *TNS3*) were hardly expressed in the individual cell lines based on qPCR analysis and were not studied further. We performed qPCR analysis to validate the remaining 17 genes. Six genes (*CDH3, ITGB1, JAK1, LAMA3, THBS1* and *TNC*) were significantly downregulated in OFC keratinocytes vs. non-OFC keratinocytes. The validation of LPP was considered not reliable due to high C_t_ values (>30). The results of the qPCR validation are provided in Figure 2. 

### 3.4. Immunostaining of Tissue Samples

**Figure 3:**
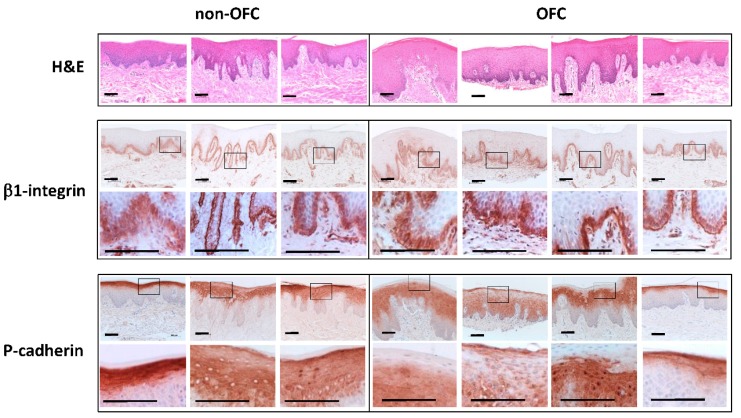
(Immuno) staining of palatal tissue samples. Tissue samples from the palatal mucosa of 3 non-OFC (left) and 4 OFC individuals (right) were sectioned and stained with Heamatoxylin–Eosin (H&E, upper panel) or for 1 integrin (middle row) or P-cadherin (lower row). 1 integrin is mainly expressed in the basal layer of the epithelium and the basal membrane. P-cadherin is mainly expressed in the upper layers of the epithelium. The bars indicate 100 m.

Tissue samples from the palate of OFC and non-OFC individuals were stained for β1 integrin and P-cadherin (*CDH3*) (Figure 3). The β1 integrin subunit was mainly expressed in the basal layer of the epithelium and in the basement membrane. No obvious differences were observed between OFC and non-OFC tissues. P-cadherin was mainly expressed in the upper layers of the epithelium excluding the cornified layer (Figure 3). Considerable variation was observed in the extent of the expression. No obvious differences were observed between OFC and non-OFC tissues.

### 3.5. Functional Validation: Scratch Assay

**Figure 4:**
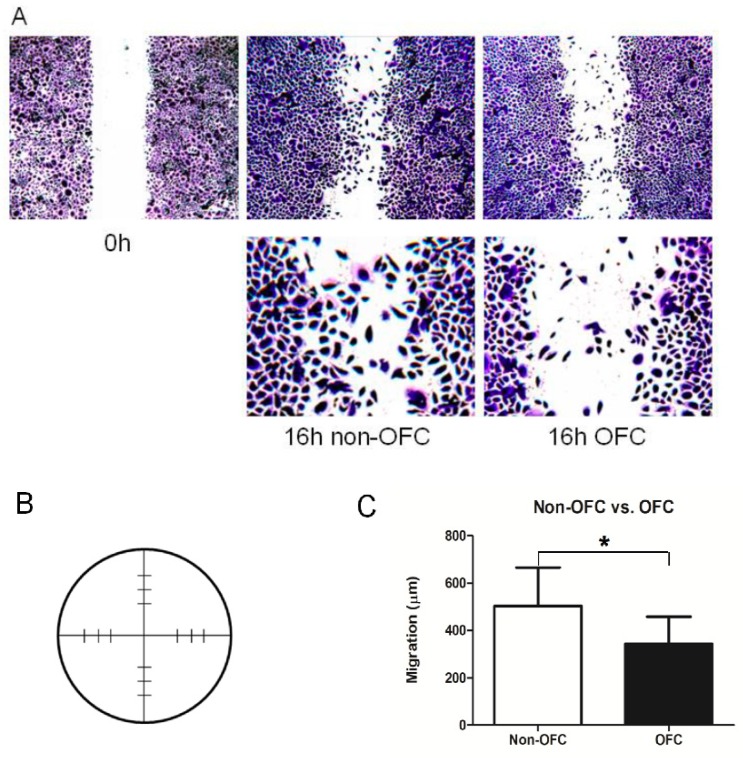
Scratch assay with palatal keratinocytes. (**A**) From left to right: Representative picture of the scratch at time point 0; non-OFC keratinocytes after 16 h; and OFC keratinocytes after 16 h. The boxed areas are enlarged. (**B**) Measurement of migration. The two perpendicular lines represent the scratches that are measured at 12 locations (short lines). (**C**) The mean migration was calculated as the mean difference between the original width of the scratch and the width after 16 h and expressed in μm. The mean migrated distance after 16 h is significantly higher in non-OFC keratinocytes vs. OFC keratinocytes (*, *p* < 0.05).

In order to analyse the impact of the downregulated genes on the migration of palatal keratinocytes, we performed a scratch assay. The migration of OFC keratinocytes (343.6 ± 29.62 μm) was significantly reduced compared to that of non-OFC keratinocytes (503.4 ± 41.81 μm) (Figure 4A). Figure 4C shows the average migration of non-OFC and OFC keratinocytes.

## 4. Discussion

To investigate possible deregulated cellular processes in OFC, we performed expression microarray analyses with pooled RNA samples from palatal keratinocytes from OFC patients and non-OFC individuals. The keratinocytes were isolated from patients of about 1.5 years of age and are obviously not identical to the embryonic cells that took part in the fusion process. However, we assume that the postnatal cells still contain some (epi) genetic traits that have contributed to the aetiology of clefting. This is corroborated by previous studies in palatal fibroblasts from patients showing remarkable differences in gene expression compared to fibroblasts from control subjects [12,13,14]. PCA of our microarray data also shows a clear difference in gene expression between OFC and non-OFC cells. GO analysis of the expression data revealed differential expression of genes involved in adhesion and migration between OFC and non-OFC keratinocytes. These data were then validated with qPCR expression analysis of individual cell lines. Finally, a scratch assay was performed to confirm the functional relevance of these findings. We found that a large part of the differentially expressed genes was linked to β1 integrin signalling and other pathways related to adhesion and migration.

Among the differentially regulated genes, several genes were related to the β1 integrin signalling pathway (*ITGB1, ITGA3, ITGB4, LAMA3, LAMC1, THBS1* and *TNC)*. Immunostaining showed that the β1 integrin subunit was similarly expressed in the basal layer of the epithelium in both OFC and non-OFC tissue samples. The gene expression of the integrin subunit β1 was significantly downregulated in OFC keratinocytes. Several β1 integrins, such as the α2β1, α3β1 and α9β1 integrins, are strongly expressed in basal keratinocytes and are involved in adhesion to the basal lamina [23]. Moreover, the ablation of β1 integrin in mouse epidermal keratinocytes strongly impairs their migration [24]. In addition, the expression of the integrin α3 subunit was slightly reduced in the microarray analysis. The integrin α3β1 regulates cell polarization and lamellipodia formation during keratinocyte migration [25]. This integrin binds to laminin 332, a major component of hemidesmosomes, which activates the focal adhesion kinase (FAK)/src pathway and the downstream Rac1 pathway [25]. The latter regulates polarization and lamellipodia formation during migration [26]. Interestingly, the expression of the laminin α3 subunit was also significantly reduced in OFC keratinocytes, which might impair laminin 332 function [27]. The expression of the laminin γ1 subunit was, however, upregulated in OFC keratinocytes. Laminin γ1 is a component of laminin 111, which is essential for basement membrane formation. Basement membrane formation is regulated by β1 integrin signalling [28]. Since the expression of the β1 integrin was significantly reduced in OFC keratinocytes, basement membrane formation might be disturbed. The integrin α9β1is known to bind to thrombospondin 1 and tenascin C, both of which are significantly reduced in OFC keratinocytes. Tenascin C is strongly expressed in migrating keratinocytes at the edge of a wound, while thrombospondin maintains the adhesion of basal keratinocytes to the basal lamina [29,30]. The above data indicate that adhesion to and migration over the basal lamina is impaired in OFC keratinocytes.

Further evidence supporting the impairment of adhesion and migration in OFC keratinocytes comes from the differential regulation of the cadherin-related genes *CDH3, JAK1* and *MLLT4.* P-cadherin immunostaining was similar in OFC and non-OFC tissue samples. The significantly reduced expression of *CDH3* in isolated OFC keratinocytes might impair intercellular adhesion and force transmission as well as polarization and lamellipodia formation. The latter processes are regulated through activation of the Cdc42 pathway [31,32,33]. Interestingly, the expression of *JAK1* was also significantly downregulated in OFC keratinocytes. Interaction of cadherins with the EGFR-activated JAK1/STAT3 signalling pathway regulates *actomyosin* contractility, which is essential for cell migration [34,35,36]. Downregulated expression of *JAK1* might hamper the activation of the JAK1/STAT3 signalling and might result in reduced adhesion and migration of OFC keratinocytes. In addition, the expression of *MLLT4* was reduced in OFC keratinocytes. Afadin 1 is an actin filament-binding protein that binds to nectin, an immunoglobulin-like cell adhesion molecule which is part of AJs together with cadherins [37]. A reduced expression of afadin 1 might disturb AJ formation. In fact, a mutation in nectin 1 *(PVRL1)* causes an autosomal recessive ectodermal dysplasia (CLPED1) characterized by OFC, syndactyly, and ectodermal dysplasia [38].

The remaining downregulated genes are either involved in formation of the focal adhesion complex (*PXN, PARVA* and *FBLIM1*) or the hemidesmosome (*DST* and *ITGB4*). Two other genes are involved in actin organisation *(MICAL2)* and the activation of keratinocyte migration *(EGFR)*. All of the abovementioned genes are involved in the regulation of adhesion and migration of keratinocytes. Hence, reduced expression of these genes probably impairs the adhesion and migration of OFC keratinocytes, which is also indicated by the scratch assay. Interestingly, it has been reported that fibroblasts from OFC patients seem to migrate faster than normal foreskin fibroblasts in a scratch assay [15], and there is increased expression of TGF. The apparent difference in migration between keratinocytes and fibroblasts of OFC patients may be due to the intrinsic migratory properties of these cells. 

In conclusion, here we show that adhesion and migration programs might be impaired in OFC keratinocytes, mainly due to reduced β1 integrin signalling and the reduced expression of several other genes. Adhesion and migration are essential for the correct formation of the secondary palate. Disruption of these processes might be induced by gene variations or epigenetic modifications that are preserved during embryogenesis and passed on to the postnatal cells. The data discussed in this study might be highly relevant to the etiopathogenesis of OFC. Yet, further research into the regulation of adhesion and migration is crucial to better understand the pathogenesis of OFC and ultimately to improve early diagnosis and prevention.

## Figures and Tables

**Table 1:** Gene expression.

GENE	Non-OFC		OFC		Fold Change	*p*
	mean	SD	mean	SD		
THBS1*	601.2	212.2	284.8	74.2	2.111	0.0057
***ARHGAP26***	289.5	59.0	156.5	45.3	1.850	0.0059
TNC*	3192	593.3	1797	234.0	1.776	0.0006
***FBLIM1***	384.9	28.0	257.2	25.8	1.496	0.0001
CDH3*	5647	476.3	3979	413.8	1.419	0.0004
***DST***	228.8	16.6	163.6	16.5	1.398	0.0003
***ITGA3***	696.1	29.8	500.5	68.4	1.391	0.0007
***EGFR***	480.9	32.6	347.1	31.2	1.385	0.0002
***ITGB4***	2554	106.0	1847	208.9	1.383	0.0003
***DOCK5***	88.24	9.1	64.72	4.5	1.363	0.0006
LAMA3*	5886	354.0	4324	817.0	1.361	0.0045
***MLLT4***	827.1	72.9	613	51.2	1.349	0.0008
***SMAD7***	126.4	10.1	95.05	9.3	1.330	0.0009
***LPP***	111.9	12.1	85.6	3.3	1.307	0.0007
ITGB1*	5418	215.1	4338	213.4	1.249	0.0001
JAK1*	196.8	8.5	157.7	13.4	1.248	0.0007
***PARVA***	419.8	42.9	342.3	11.5	1.226	0.0026
***PXN***	650.7	25.4	537.8	53.4	1.210	0.0031
***MICAL2***	42.61	5.1	35.71	3.4	1.193	0.0330
***LAMC1***	1263	42.0	1066	84.2	1.185	0.0026
***TNS3***	101.9	31.7	98.31	44.8	1.037	0.7275

Mean non-OFC and mean OFC represent average relative expression in non-OFC and OFC cells, respectively. *, statistically significant differentially expressed genes validated by RT-qPCR.

## Data Availability

Microarray data have been deposited in the GEO database (Accession No. GSE139222): https://www.ncbi.nlm.nih.gov/geo/query/acc.cgi?acc=GSE139222.

## Supplementary Materials

The following are available online at https://www.mdpi.com/2073-4425/10/11/836/s1, Table S1: SB_DAVID_GO_CC_0001.

## References

[1] Tanaka S.A., Mahabir R.C., Jupiter D.C., Menezes J.M. (2012). Updating the epidemiology of cleft lip with or without cleft palate. Plast. Reconstr. Surg..

[2] Dixon M.J., Marazita M.L., Beaty T.H., Murray J.C. (2011). Cleft lip and palate: Understanding genetic and environmental influences. Nat. Rev. Genet..

[3] Lan Y., Xu J., Jiang R. (2015). Cellular and molecular mechanisms of palatogenesis. Curr. Top. Dev. Biol..

[4] Chai Y., Maxson R.E. (2006). Recent advances in craniofacial morphogenesis. Dev. Dyn..

[5] Richardson R.J., Hammond N.L., Coulombe P.A., Saloranta C., Nousiainen H.O., Salonen R., Berry A., Hanley N., Headon D., Karikoski R. (2014). Periderm prevents pathological epithelial adhesions during embryogenesis. J. Clin. Investig..

[6] Hu L., Liu J., Li Z., Ozturk F., Gurumurthy C., Romano R.A., Sinha S., Nawshad A. (2015). TGFbeta3 regulates periderm removal through DeltaNp63 in the developing palate. J. Cell. Physiol..

[7] Yoshida M., Shimono Y., Togashi H., Matsuzaki K., Miyoshi J., Mizoguchi A., Komori T., Takai Y. (2012). Periderm cells covering palatal shelves have tight junctions and their desquamation reduces the polarity of palatal shelf epithelial cells in palatogenesis. Genes Cells Devoted Mol. Cell. Mech..

[8] Cuervo R., Covarrubias L. (2004). Death is the major fate of medial edge epithelial cells and the cause of basal lamina degradation during palatogenesis. Development.

[9] Vaziri Sani F., Hallberg K., Harfe B.D., McMahon A.P., Linde A., Gritli-Linde A. (2005). Fate-mapping of the epithelial seam during palatal fusion rules out epithelial-mesenchymal transformation. Dev. Biol..

[10] Kim S., Lewis A.E., Singh V., Ma X., Adelstein R., Bush J.O. (2015). Convergence and extrusion are required for normal fusion of the mammalian secondary palate. PLoS Biol..

[11] Palecek S.P., Loftus J.C., Ginsberg M.H., Lauffenburger D.A., Horwitz A.F. (1997). Integrin-ligand binding properties govern cell migration speed through cell-substratum adhesiveness. Nature.

[12] Bodo M., Baroni T., Carinci F., Becchetti E., Bellucci C., Pezzetti E., Conte C., Evangelisti R., Carinci P. (1999). TGFbeta isoforms and decorin gene expression are modified in fibroblasts obtained from non-syndromic cleft lip and palate subjects. J. Dent. Res..

[13] Baroni T., Bellucci C., Lilli C., Pezzetti F., Carinci F., Becchetti E., Carinci P., Stabellini G., Calvitti M., Lumare E. (2006). Retinoic acid, GABA-ergic, and TGF-beta signaling systems are involved in human cleft palate fibroblast phenotype. Mol. Med..

[14] Bosi G., Evangelisti R., Valeno V., Carinci F., Pezzetti F., Calastrini C., Bodo M., Carinci P. (1998). Diphenyihydantoin affects glycosaminoglycans and collagen production by human fibroblasts from cleft palate patients. J. Dent. Res..

[15] Beyeler J., Schnyder I., Katsaros C., Chiquet M. (2014). Accelerated wound closure in vitro by fibroblasts from a subgroup of cleft lip/palate patients: Role of transforming growth factor-alpha. PLoS ONE.

[16] Liu J., Lamme E.N., Steegers-Theunissen R.P.M., Krapels I.P.C., Bian Z., Marres H., Spauwen P.H.M., Kuijpers-Jagtman A.M., Von den Hoff J.W. (2008). Cleft Palate Cells Can Regenerate a Palatal Mucosa In Vitro. J. Dent. Res..

[17] Jansen B.J., Gilissen C., Roelofs H., Schaap-Oziemlak A., Veltman J.A., Raymakers R.A., Jansen J.H., Kogler G., Figdor C.G., Torensma R. (2010). Functional differences between mesenchymal stem cell populations are reflected by their transcriptome. Stem Cells Dev..

[18] Huang D.W., Sherman B.T., Lempicki R.A. (2009). Bioinformatics enrichment tools: Paths toward the comprehensive functional analysis of large gene lists. Nucleic Acids Res..

[19] Huang da W., Sherman B.T., Lempicki R.A. (2009). Systematic and integrative analysis of large gene lists using DAVID bioinformatics resources. Nat. Protoc..

[20] de Jongh G.J., Zeeuwen P.L.J.M., Kucharekova M., Rolph Pfundt R., van der Valk P.G., Blokx W., Dogan A., Hiemstra P.S., van de Kerkhof P.C., Schalkwijk J. (2005). High expression levels of keratinocyte antimicrobial proteins in psoriasis compared with atopic dermatitis. J. Investig. Dermatol..

[21] Aerts S., Lambrechts D., Maity S., Van Loo P., Coessens B., De Smet F., Tranchevent L.C., De Moor B., Marynen P., Hassan B. (2006). Gene prioritization through genomic data fusion. Nat. Biotechnol..

[22] Tranchevent L.C., Barriot R., Yu S., Van Vooren S., Van Loo P., Coessens B., De Moor B., Aerts S., Moreau Y. (2008). ENDEAVOUR update: A web resource for gene prioritization in multiple species. Nucleic Acids Res..

[23] DiPersio C.M., Zheng R., Kenney J., Van De Water L. (2016). Integrin-mediated regulation of epidermal wound functions. Cell Tissue Res..

[24] Grose R., Hutter C., Bloch W., Thorey I., Watt F.M., Fässler R., Brakebusch C., Werner S. (2002). A crucial role of β1 integrins for keratinocyte migration in vitro and during cutaneous wound repair. Development.

[25] Choma D.P., Milano V., Pumiglia K.M., DiPersio C.M. (2007). Integrin alpha3beta1-dependent activation of FAK/Src regulates Rac1-mediated keratinocyte polarization on laminin-5. J. Investig. Dermatol..

[26] Etienne-Manneville S., Alan Hall A. (2002). Rho GTPases in cell biology. Nature.

[27] Kiritsi D., Has C., Bruckner-Tuderman L. (2013). Laminin 332 in junctional epidermolysis bullosa. Cell Adhes. Migr..

[28] Aumailley M., Pesch M., Tunggal L., Gaill F., Fässler R. (2000). Altered synthesis of laminin 1 and absence of basement membrane component deposition in β1 integrin-deficient embryoid bodies. J. Cell Sci..

[29] Häkkinen L., Hildebrand H.C., Berndt A., Kosmehl H., Larjava H. (2000). Immunolocalization of tenascin-c, a9 integrin subunit, and avb6 integrin during wound healing in human oral mucosa. J. Histochem. Cytochem..

[30] Sweetwyne M.T., Murphy-Ullrich J.E. (2012). Thrombospondin1 in tissue repair and fibrosis: TGF-beta-dependent and independent mechanisms. Matrix Biol. J. Int. Soc. Matrix Biol..

[31] Plutoni C., Bazellieres E., Le Borgne-Rochet M., Comunale F., Brugues A., Seveno M., Planchon D., Thuault S., Morin N., Bodin S. (2016). P-cadherin promotes collective cell migration via a Cdc42-mediated increase in mechanical forces. J. Cell Biol..

[32] Bazellieres E., Conte V., Elosegui-Artola A., Serra-Picamal X., Bintanel-Morcillo M., Roca-Cusachs P., Munoz J.J., Sales-Pardo M., Guimera R., Trepat X. (2015). Control of cell-cell forces and collective cell dynamics by the intercellular adhesome. Nat. Cell Biol..

[33] Ng M.R., Besser A., Danuser G., Brugge J.S. (2012). Substrate stiffness regulates cadherin-dependent collective migration through myosin-II contractility. J. Cell Biol..

[34] Geletu M., Guy S., Arulanandam R., Feracci H., Raptis L. (2013). Engaged for survival: From cadherin ligation to STAT3 activation. Jak-Stat.

[35] Ridley A.J., Schwartz M.A., Burridge K., Firtel R.A., Ginsberg M.H., Borisy G., Parsons J.T., Horwitz A.R. (2003). Cell migration: Integrating signals from front to back. Science.

[36] Sanz-Moreno V., Gaggioli C., Yeo M., Albrengues J., Wallberg F., Viros A., Hooper S., Mitter R., Feral C.C., Cook M. (2011). ROCK and JAK1 signaling cooperate to control actomyosin contractility in tumor cells and stroma. Cancer Cell.

[37] Ikeda W., Nakanishi H., Miyoshi J., Mandai K., Ishizaki H., Tanaka M., Togawa A., Takahashi K., Nishioka H., Yoshida H. (1999). Afadin: A key molecule essential for structural organization of cell–cell junctions of polarized epithelia during embryogenesis. J. Cell Biol..

[38] Suzuki K., Hu D., Bustos T., Zlotogora J., Richieri-Costa A., Helms J.A., Spritz R.A. (2000). Mutations of PVRL1, encoding a cell-cell adhesion molecule/herpesvirus receptor, in cleft lip/palateectodermal dysplasia. Nat. Genet..

